# Supplementary material for: Effect of sunflower seed oil emollient therapy on newborn infant survival in Uttar Pradesh, India: A community-based, cluster randomized, open-label controlled trial
Source: PLoS Med. 2021 Sep 28;18(9):e1003680. doi: 10.1371/journal.pmed.1003680 (PMC8478176; doi:10.1371/journal.pmed.1003680)
Supplement: S1 Analysis Plan — (DOCX) [file pmed.1003680.s002.docx]

**Statistical methods**

Analysis will be done by intention-to-treat. Participants who were residing in a particular cluster at the time of enrolment into the study will be analyzed as part of that cluster. Point estimates (summary statistics) of outcome measures for each study arm will be calculated as the mean of cluster values, giving an equal weight to each cluster.

For binary outcomes^[[1]](#endnote-1)^ (mortality, incidence, prevalence), the overall risk/rate for each cluster will be calculated. Risks/rates for each cluster will be shown, by strata and arm. A log transformation using Taylor series approximation will be applied to the risk/rate for each cluster for normalization. The mean and SD of these log risks/rates will be used to obtain the geometric mean and associated 95% CI for the intervention arm of the study. Linear regression of the log mean risk/rate on strata and arm will be used to estimate the relative risk/rate and 95% CI associated with the intervention. The approximate variance for the mean risks/rates ratio will be obtained based on the residual mean square from a twoway analysis of variance (ANOVA) of cluster log-risk/rate on stratum and study arm. A 95% CI for the relative risk/rate will be calculated from this variance using a t-statistic.

For continuous outcomes^[[2]](#endnote-2)^ such as birth weight measurements, the overall mean for each cluster will be calculated, and means for each cluster will be shown by strata and arm. The arithmetic mean and SD of these mean scores/numbers and associated 95% CI for the intervention arm of the study will be calculated. Linear regression of the mean score/number on strata and arm (2-way ANOVA on intervention and strata) will be used to estimate the difference in score/number and 95% CI associated with the intervention.

The results obtained in the intervention arm will be compared against the control arm. In case of any imbalance in the study arms identified post randomization; the DSMB will recommend adjustments to the analysis at its initial meeting.

Sub-analyses^[[3]](#endnote-3)^ are planned for premature/full-term, low birth weight/normal birth weight, facility/ home births, wealth quintiles and number of visitations/emollient use (dose). In addition to the above, cluster-level secondary per protocol analyses will be conducted to study only those babies who used oil as envisaged with their outcomes, comparing to those in control clusters.

1. We diverged from this method during the final analysis as we had not factored in the wide difference in cluster sizes in our pre-specified analysis plan. As per Hayes & Moulton in Cluster Randomised Trials (2nd ed., 2017), when clusters are of widely varying sizes, the basic assumption of an unpaired t-test of cluster summary measures being identically distributed with equal variance is invalidated, and greater power and precision would result from weighting the analysis to taken into account the different variances. They further recommend that for larger than 15-20 clusters, individual-level regression is more efficient than the simpler two-stage methods, especially when cluster sizes vary substantially. We consulted a senior biostatistician at Stanford University, Prof. Lu Tian, who had never been involved in this study and had no access to the data, on a suitable analytical approach, prior to initiating the primary analysis. He advised us on our current analytical approach of individual-level analysis adjusted for within-cluster correlation using a random-effects logistic regression model. We were further advised to adjust for risk factors associated with SES (caste), delivery factors (birth attendant/delivery place), maternal factors (age, education, gravidity/parity), and infant factors (birth weight, multiple births, sex). The same approach was used for all analysis involving binary outcomes, including secondary analysis. [↑](#endnote-ref-1)
2. We have not conducted any statistical tests involving continuous outcomes in this manuscript. [↑](#endnote-ref-2)
3. We conducted sub-analyses as planned. As LMP dates were not reliable, we were unable to conduct the sub-analysis based on gestational age, and instead chose very low birth weight infants (<=1500g) as a proxy for infants most vulnerable to skin barrier disruption. [↑](#endnote-ref-3)
