# Supplementary material for: Effect of sunflower seed oil emollient therapy on newborn infant survival in Uttar Pradesh, India: A community-based, cluster randomized, open-label controlled trial
Source: PLoS Med. 2021 Sep 28;18(9):e1003680. doi: 10.1371/journal.pmed.1003680 (PMC8478176; doi:10.1371/journal.pmed.1003680)
Supplement: Attachment — Submitted filename: Manuscript PMEDICINE-D-20-04871R1.docx [file pmed.1003680.s003.docx]

Manuscript PMEDICINE-D-20-04871R1: Impact of emollient therapy with sunflower seed oil on survival of newborn infants in Uttar Pradesh, India: a community-based, cluster randomized, open-label controlled trial.

Reviewer Comments

Overall

This manuscript addresses a timely and extremely important topic, namely the effect of emollient therapy on mortality within the neonatal period and in a setting with a high mortality rate. Efforts to reduce neonatal mortality remain paramount and large scale, randomized trials such as this one are required to determine treatment effectiveness. Additionally, the work extends previous reports of the benefits of treatment with sunflower seed oil on infection and mortality among premature infants in a hospital setting. The authors appropriately conducted a sub-analysis of the effects on infants < 1500 grams, who are at increased risk of infection due to an underdeveloped skin barrier. The design goal was to develop information that would be generalizable to other settings with a focus of reducing neonatal mortality.

The comments below are intended to clarify aspects of each section.

Methods

1. Delivery of sunflower seed oil intervention. Previous studies in hospital settings used gentle massage. The intervention used a gentle massage, taught to families who received it. However, the control arm conducted massage as usual, namely a vigorous massage. Therefore, the two arms differed in oil type and in massage process. Is there information regarding massage type/vigor that can be cited? What are the potential effects of the difference in massage on the outcomes?
2. Please provide information about the environmental conditions (temperature, relative humidity) throughout the study and in the two study arms and indicate whether these factors impacted the results or caused the study groups to be different.
3. Enrollment within 7 days of delivery: Please provide detailed information about the mean time after birth until enrollment. From the description, it seems possible that infants on the sunflower seed oil intervention were massaged 3-5 times daily for 7 days with the usual oil. This would be a confounder as infants would have been exposed to two treatments within the first 29 days of life. How could this confounder be addressed?

Is it possible to look at only those infants who began the intervention or control within 1-2 days of birth as a subset to remove this confounder? The rate of change in skin barrier adaptation following birth is rapid enough that treatment with a different oil for the first few days could confound the results? Perhaps there are sufficient numbers to address this question.

Similarly, is it possible to look only at the premature infants (<1500 g) who began the intervention or control within 1-2 days of birth to remove the confounder of use of another emollient for routine massage before enrollment?

4. Please provide ore detail on the composition of SSO as this will enable readers to compare this study with others in the literature. What is meant by absence of harmful ingredients? What was the variability throughout the study?
5. Is there any potential effect on the behavior of the participants who received instruction on SSO application that was not experienced by the control group? Is it possible that there was a difference in number of massages per day or amount of oil used for the intervention?
6. Was gestational age information collected? If so, please report it for the various groups listed in Tables 1 and 2.
7. Please clarify the process that was used to document the use of the oil type in both arms. How was it determined that SSO was used exclusively by 30.4% of subjects and mustard oil by 36%? What is the expected reliability of this method?
8. The measurement of weight approximated birthweight, as indicated. Were there records for those who delivered with a professional that would have birthweight? Discuss how the weight at enrollment impacted the design or analysis, i.e., how would weight loss or gain between birth and start of study procedures (for intervention) impact outcome and be addressed?

Results

1. Please provide demographic information for the groups in table 2, including the GA, weights, gender distribution, day of life at enrollment and statistical comparison. Table 2 currently lists the primary outcome only.
2. Is there value in showing the time from birth to neonatal death for the two groups?
3. There were cases of skin rash, etc., for each group. Please provide more detail on the nature of these conditions. Were they associated with a particular birthweight or GA?
4. Given the importance of skin barrier integrity in neonatal infection, provide the rationale for not including skin assessment as a measure of emollient effects on skin. Skin rash has been reported previously among newborns receiving either sunflower seed oil or mustard oil in a rural community setting (Summers A, et al. J Perinatol. 2018 Jan;38(1):64-70. doi: 10.1038/jp.2017.158 and Summers A, et al. BMC Pediatr. 2019 Dec 23;19(1):512. doi: 10.1186/s12887-019-1871-2). Was rash or miliaria observed in subjects of the present study? How did the skin integrity in the present study compare to previous reports?
5. In Table 2, section C, please specify the symbol in the title (§) as it is not currently defined.

Discussion

1. On page 20, in the paragraph beginning in line 400, include a discussion of the information in the previous report by Summers, A., et al, that describes the effect of mustard versus sunflower seed oil for routine repetitive neonatal skin massage (Summers A, et al. BMC Pediatr. 2019 Dec 23;19(1):512. doi: 10.1186/s12887-019-1871-2) Summers A, et al. BMC Pediatr. 2019 Dec 23;19(1):512. doi: 10.1186/s12887-019-1871-2.
2. Given that mustard oil was used exclusively 36% of the time in the control group, what was the usage of mustard oil exclusively in the intervention group and how would this impact the conclusions?
3. What were the “other” oils used by subjects in either arm when they did not use mustard oil or sunflower seed oil? Is it possible to comment on the range of oils used, along with their expected compositions? How much variation in fatty acid content of various types is to be expected within this population and/or region in India?
4. What is the reason for the increase in exclusive adherence from 15.2% to 38.7% later in the study?
5. On page 21, in the paragraph starting with line 430, the authors discuss that the study population who were subject to mortality due to skin barrier compromise may have been too low. What is the basis for this statement? If there is information about the relationship between birthweight or gestational age, day of life, skin barrier integrity/compromise and mortality due to compromise, please elaborate in this section and include the references. Similarly, provide information about the percent of the population that was considered normal term intra-uterine growth restricted. It would be of interest to know more details about the two populations in terms of growth restriction and percentage of infants at various weights or gestational ages.
6. On page 21, in lines 439-441, there is a statement that mortality was 52% reduced in infants ≤ 1500 g, despite low adherence to SSO. What are the potential explanations for this finding? Does the statement indicate that the mortality was reduced enough in the infants who adhered to the treatment to account for the reduction in the total group? Please clarify.
7. On page 24, in the paragraph starting with 505, include a discussion of the effects of mustard oil massage in the immediate newborn period in Nepal.
