# Supplementary material for: Effect of sunflower seed oil emollient therapy on newborn infant survival in Uttar Pradesh, India: A community-based, cluster randomized, open-label controlled trial
Source: PLoS Med. 2021 Sep 28;18(9):e1003680. doi: 10.1371/journal.pmed.1003680 (PMC8478176; doi:10.1371/journal.pmed.1003680)
Supplement: Attachment — Submitted filename: Responses_PLOS Medicine_21_Dec_2020.docx [file pmed.1003680.s004.docx]

**PMEDICINE-D-20-04871R1: Impact of emollient therapy with sunflower seed oil on survival of newborn infants in Uttar Pradesh, India: a community-based, cluster randomized, open-label controlled trial**

**Responses to Editors’ and Reviewers’ Comments**

**Requests from the editors:**

1.	Please address the reviewers’ comments below.

**Response**: Thank you very much for the extremely helpful comments from the editors and reviewers. Please find point-by-point responses below.

2.	Financial Disclosure: Please add the following, if true (otherwise please clarify): “The funders had no role in study design, data collection and analysis, decision to publish, or preparation of the manuscript.”

**Response**: The following statement was in the original submission, in the section, Role of the funding source: “The funder had no role in data analysis, data interpretation, or writing of the manuscript.” This statement was revised to: “*The funder had no role in study design, data collection and analysis, decision to publish, or preparation of the manuscript.*”

3.	Data Availability: We recommend that you deposit your data to a more permanent repository than Dropbox. Some options (most are free of charge) can be found here: <https://journals.plos.org/plosmedicine/s/recommended-repositories>.

**Response**: This has now been shared at <https://doi.org/10.7910/DVN/TGNC9H> on the Harvard Dataverse repository.

4.	Abstract:

a.	Please include trial recruitment dates.

**Response**: The words in *italics* were added: “…were enrolled *from November 2014 to October 2016.”*

b.	Around line 50: Please describe the primary and the main secondary objectives.

**Response**: The words in *italics* were added: “*Primary analysis was by* intention-to-treat with NMR *and post-24-hour NMR* as the primary outcomes. *Secondary analysis included per-protocol analysis and* sub-group analyses for NMR.”

c.	Please provide summary participant demographics, eg. sex.

**Response**: The words in *italics* were added: We enrolled 13,478 *(52.2% male, mean weight 2575.0 grams ± 521.0)* and 13,109 *(52.0% male, mean* weight 2607.0 g ± 509.0*)* newborn infants in the intervention and comparison clusters, respectively.

d.	Please include a description of any adverse events, and whether they were attributed to the study.

**Response**: The following sentence was added: “*No severe adverse events were attributable to the intervention.*”

e.	In the last sentence of the Abstract Methods and Findings section, please describe the main limitation(s) of the study's methodology.

**Response**: The following sentence was added: “*The study was limited by inability to mask allocation to study workers or participants, and by measurement of emollient use based on caregiver responses and not actual observation.”*

f.	Line 65: Please start with “In this trial, we observed that…”

**Response**: This has been updated.

5.	At this stage, we ask that you include a short, non-technical Author Summary of your research to make findings accessible to a wide audience that includes both scientists and non-scientists. The Author Summary should immediately follow the Abstract in your revised manuscript. This text is subject to editorial change and should be distinct from the scientific abstract. Please see our author guidelines for more information: <https://journals.plos.org/plosmedicine/s/revising-your-manuscript#loc-author-summary>

**Response**: An Author Summary was added as follows:

# ****Why Was This Study Done?****

# Preterm infants have an immature skin barrier, which makes them susceptible to further skin barrier disruption and increased systemic invasion by pathogens.

# Studies in animal models under laboratory conditions have found that emollients differ in how they affect skin barrier integrity, with some emollients, such as sunflower seed oil (SSO) containing high concentrations of linoleic acid and having barrier enhancing properties, and some others, such as mustard oil, associated with barrier impairment.

# A meta-analysis of three trials from low-resource settings in South Asia and the Middle East evaluating effects of emollient therapy vs. no massage in hospitalized preterm infants showed a 50% reduction in bloodstream-proven infections and 27% reduction in neonatal mortality rate (NMR).

# In high-NMR community settings in low- and middle-income countries, high-risk skin care practices such as vigorous removal of vernix at birth, near-universal vigorous massage, poor hygiene and high environmental load of pathogens have been observed. We hypothesized that in these settings, even term and normal birth weight babies may be susceptible to impaired skin barrier integrity, and therefore could be at increased risk of mortality.

# To our knowledge, this is the first-ever study of emollient therapy by families in a community setting, with usual skin care practice as the comparison, and with an aim to evaluate population-based reduction in neonatal mortality rate (NMR). Crucially, this study compares improved massage with a skin-barrier enhancing oil (SSO) against usual massage practices typically with mustard oil, in contrast to previous hospital studies that compared emollient use vs. no massage. We also emphasized initiation of SSO use in interventions infants immediately after birth.

**What Did the Researchers Do and Find?**

- We conducted a cluster randomized controlled open-label trial, randomizing 276 villages equally to intervention or control. Three times daily gentle applications of high-linoleate sunflower seed oil (SSO) were promoted in intervention clusters for all newborns as compared to usual massage practices in the control arm.
- The trial enrolled 13,478 and 13,109 infants in intervention and control clusters, respectively. Primary analysis was conducted according to intervention group assignment (intention-to-treat) and supplemented by successful adherence to recommended treatment (per protocol). We also repeated the analysis for sub-groups of newborns that may respond differentially to treatment.
- SSO therapy when promoted universally for all newborns was not effective in reducing NMR. Acceptance of SSO was high at 89.3%, but adherence to recommended exclusive SSO application overall was low at 30.4%.
- Per-protocol analysis found 58% significantly lower neonatal mortality in intervention infants who were treated exclusively with SSO as recommended compared to infants in the comparison clusters who exclusively received mustard oil massage.
- Sub-group analyses found a significant 52% reduction in NMR in very low birth weight infants (≤ 1500 g) who were at highest risk of compromised skin barrier function, but no mortality impact in subgroups of infants born in health facilities, at home, or with low birth weight (< 2500 g).

**What Do These Findings Mean?**

- We do not recommend universal promotion of SSO therapy under typical implementation conditions for mortality reduction.
- Our data showing low levels of adherence to recommended treatment and a significant mortality reduction by per protocol analysis indicates that potential benefit of SSO over mustard oil cannot be ruled out and needs systematic investigation.
- Results from our sub-group analysis on the highest-risk category of very low birth weight infants are consistent with previous hospital-based studies and potentially extend the applicability of emollient therapy in this sub-group to the facility-community continuum.
- Further research is recommended to evaluate the applicability of emollient therapy along the facility-community continuum directed towards newborn infants with highest risk of compromised skin barrier function.

6.	Please use the "Vancouver" style for reference formatting, and see our website for other reference guidelines <https://journals.plos.org/plosmedicine/s/submission-guidelines#loc-references>. Citations should be in square brackets and not superscript.

**Response**: Reference citations were changed from superscripts to square brackets.

7.	Line 173: Please clarify whether written informed consent for trial participation was obtained from parents/guardians.

**Response**: This is clarified with the addition of the following sentences in the Ethics approval section of the Methods: “*Community-level consent was obtained from community leaders in each of the study clusters, and written informed consent was obtained from the mothers/guardians of all infants prior to enrolment*.”

8.	Lines 301-307: Please remove funding details here – this should be included on the online submission form instead.

**Response**: Done.

9.	Line 385: Please clarify: “This is the first community-based trial, to our knowledge, to report…”

**Response**: The phrase “…*to our knowledge*…” was added.

10.	Lines 537-568: Please delete these sections here - they should be included on the online submission form instead.

**Response**: This has been done as suggested.

11.	Figure 2 legend: Please rename “Participant flow diagram”.

**Response**: Done.

12.	Please complete the CONSORT checklist, include it as a Supporting Information file, and ensure that all components of CONSORT are present in the manuscript. When completing the checklist, please use section and paragraph numbers, rather than page numbers.

**Response**: We have revised and resubmitted the CONSORT checklist as suggested.

13.	Please include the trial protocol as a Supporting Information file, and add a citation to it in the Methods.

**Response**: The protocol is attached as a Supporting Information file, and it is cited in the Methods and in the References as follows: “*The study was designed as a two-arm cluster-randomized controlled efficacy trial [16].”* We have also made the protocol available at the same location as the dataset at <https://doi.org/10.7910/DVN/TGNC9H> on the Harvard Dataverse repository.

Reference 16.	Kumar V, Kumar A. Impact of topical application of cold-pressed sunflower seed oil with improved massage practices on neonatal mortality: a cluster randomized controlled trial in rural North India, May 14, 2014. Accessible at: https://doi.org/10.7910/DVN/TGNC9H, Harvard Dataverse, V1; WHO_Emollient_protocol_14May2014_v2.0.pdf [fileName]

**Comments from the reviewers:**

**Reviewer #1**: Please see the attached word document.

Overall

This manuscript addresses a timely and extremely important topic, namely the effect of emollient therapy on mortality within the neonatal period and in a setting with a high mortality rate. Efforts to reduce neonatal mortality remain paramount and large scale, randomized trials such as this one are required to determine treatment effectiveness. Additionally, the work extends previous reports of the benefits of treatment with sunflower seed oil on infection and mortality among premature infants in a hospital setting. The authors appropriately conducted a sub-analysis of the effects on infants < 1500 grams, who are at increased risk of infection due to an underdeveloped skin barrier. The design goal was to develop information that would be generalizable to other settings with a focus of reducing neonatal mortality.

The comments below are intended to clarify aspects of each section.

Methods

1. Delivery of sunflower seed oil intervention. Previous studies in hospital settings used gentle massage. The intervention used a gentle massage, taught to families who received it. However, the control arm conducted massage as usual, namely a vigorous massage. Therefore, the two arms differed in oil type and in massage process. Is there information regarding massage type/vigor that can be cited? What are the potential effects of the difference in massage on the outcomes?

**Response**: The trial was designed to test a package of sunflower seed oil (SSO) plus gentle massage. The required sample size to separately test the contributions of SSO and massage was prohibitively large. Given the size and scale of the study, it was also not possible to directly observe massage and skincare practices for all newborns, and respondent recall was the primary source of information. Given the considerable variance in how respondents perceive their massage technique in terms of how gentle it was, we found it best to avoid questions on massage technique. The following text in italics has been added to the Discussion as follows: “The mechanism of impact of SSO therapy on newborn survival is biologically plausible and may be multi-factorial, but requires further research [12,29,30]. *The intervention was promoted as a package of SSO plus gentle massage, and thus, we cannot disentangle the separate impacts of the oil from the way in which it was applied. However, it is likely that they interact and that both are important in intervention promotion [29].* Evidence *furthermore* suggests that emollient therapy has benefits for very preterm infants beyond that provided by massage alone [31-33].

2. Please provide information about the environmental conditions (temperature, relative humidity) throughout the study and in the two study arms and indicate whether these factors impacted the results or caused the study groups to be different.

**Response**: Temperature and humidity are unlikely to have impacted trial outcomes, as the enrolment rate was similar across both the arms throughout the study – please see table below:

| **Season** | **Intention-to-treat** | | | | **Per protocol** | | | |
| --- | --- | --- | --- | --- | --- | --- | --- | --- |
|  | **Intervention** | | **Control** | | **Intervention** | | **Control** | |
|  | Freq | % | Freq | % | Freq | % | Freq | % |
| Dec-Feb | 2091 | 15.5 | 1904 | 14.5 | 559 | 13.7 | 693 | 14.7 |
| Mar-May | 2405 | 17.8 | 2419 | 18.5 | 827 | 20.2 | 887 | 18.8 |
| Jun-Aug | 4892 | 36.3 | 4819 | 36.8 | 1489 | 36.4 | 1818 | 38.5 |
| Sep-Nov | 4090 | 30.4 | 3967 | 30.3 | 1221 | 29.8 | 1322 | 28.0 |

Average monthly temperature, rainfall and relative humidity in Raebareli, India, can be found at this site: https://weatherspark.com/y/110425/Average-Weather-in-Raebareli-India-Year-Round. We examined treatment adherence by season of the year, according to four seasons (December-February, March-May, June-August, September-November). Despite the wide seasonal variation in environmental condition, variation in treatment adherence across seasons was small, ranging from 33.2 to 37.6% for exclusive use of mustard oil in the comparison arm, and 27.3 to 34.1% for exclusive use of SSO in the intervention arm. The following text in italics was added to the manuscript: “In the intervention arm, 4096 infants received SSO therapy exclusively, an adherence rate of 30.4%, *and* in the comparison arm, 4720 infants (36.0%) received mustard oil exclusively, *with limited seasonal variation*.”

3. Enrollment within 7 days of delivery: Please provide detailed information about the mean time after birth until enrollment. From the description, it seems possible that infants on the sunflower seed oil intervention were massaged 3-5 times daily for 7 days with the usual oil. This would be a confounder as infants would have been exposed to two treatments within the first 29 days of life. How could this confounder be addressed?

**Response**: Following is detailed information about time to the first visit after birth (enrollment) *–* for subjects overall in the intention-to-treat analysis and in the per-protocol analysis *–* which is also when the first data collection took place, including weighing. Table 1 reports the mean age and weight at the time of enrollment – these were similar in the groups that were compared. The following text in italics was added to the Results: “*At the first postnatal (enrolment) visit, that occurred on day 2 ± 2 for both intervention and comparison arms (Table 1)*, 589 infants (2.2%) weighed ≤1500 g.”

The confounder with respect to differences in early exposure to the treatments cannot be addressed in the intent-to-treat analysis by definition, but has been completely addressed in the per-protocol analysis. At the time of the first visit, participants were asked about emollient application practices from the time of birth until then; this included the products applied but not the number of times per day that they were applied. During the antenatal visit, families in intervention clusters had been counseled on the importance of early initiation of SSO therapy and provided SSO for use immediately after delivery and for the first few days. Thus, we were able to obtain a retrospective record of SSO or mustard oil use going back to the time of birth. This information was used to define per protocol use as described in the Methods.

Is it possible to look at only those infants who began the intervention or control within 1-2 days of birth as a subset to remove this confounder? The rate of change in skin barrier adaptation following birth is rapid enough that treatment with a different oil for the first few days could confound the results? Perhaps there are sufficient numbers to address this question.

**Response**: This may not be needed, as the per protocol analysis is already designed to answer this question, as it considers oil use from the first cleaning of the newborn infant, generally within minutes after birth, and compares intervention infants who exclusively received SSO from birth to comparison infants who exclusively received mustard oil from birth. This has been clarified in the Methods section on the per protocol analysis with the addition of the text in italics: “Complete adherence to SSO was defined as using SSO to initially cleanse the newborn infant *within minutes of birth*, applying SSO at the first application during the first 6 hours after delivery, and subsequently applying SSO exclusively during the entire follow-up period.” This analysis shows that among infants in the intervention arm who received exclusive SSO from the time of the first cleansing (essentially from birth), neonatal mortality was reduced by 58% (OR 0.42, 95% CI 0.31-0.58, p<0.01) compared to infants in the comparison arm who exclusively received mustard oil applications from the time of birth. When we examined neonatal mortality at the time of the first visit at a median time of about 2 days after birth, we also found a significant reduction in mortality (OR 0.57, 95% CI 0.43-0.76, p<0.01) among infants in the intervention arm who received exclusive SSO from the time of the first cleansing compared to infants in the comparison arm who exclusively received mustard oil applications from the time of the first cleansing. This suggests that any impact of SSO in reducing mortality was seen early, which is also reflected in the survival curve from the per protocol analysis (Figure 4A). The following text was added to the per protocol Results: “*Survival analysis suggests an early benefit in intervention infants treated exclusively with SSO, primarily in the first week after birth but continuing to accrue throughout the neonatal period (Figure 4A).”*

Similarly, is it possible to look only at the premature infants (<1500 g) who began the intervention or control within 1-2 days of birth to remove the confounder of use of another emollient for routine massage before enrollment?

**Response**: We did not conduct per protocol sub-group analysis on very low birth weight infants as the sample size of premature infants (<1500g) is already low (n=278 for control and n=311 for intervention) and it would be unadvisable to break it up further.

4. Please provide more detail on the composition of SSO as this will enable readers to compare this study with others in the literature. What is meant by absence of harmful ingredients? What was the variability throughout the study?

**Response**: Each batch of SSO received from our supplier was sent to an internationally accredited laboratory (SGS India Private Limited based in Gurgaon, India) that performs food certifications amongst a variety of services offered. The procured cold-pressed SSO was tested based on the lab’s protocols for fatty acid composition, banned chemicals (agaric acid, hydrocyanic acid, hypericine and saffrole), aflatoxins, heavy metals and pesticides. This was done to ensure that the oil had the desired fatty acid composition with ≥60% linoleic acid, and also met food safety standards to ensure to eliminate the possibility of any harm. Between 2-Feb-2015 and 28-Apr-2016, eight batches of SSO were approved for use based on test reports, with linoleic acid content as mean 61.5%, stdev 1.6%, range (59.8 – 64.6). The following text was added: ). “*SSO composition was tested for each of the eight batches of oil procured to ensure that they met food safety standards; there was minimal variation in linoleic acid content (61.5% ± 1.6%).“*

5. Is there any potential effect on the behavior of the participants who received instruction on SSO application that was not experienced by the control group? Is it possible that there was a difference in number of massages per day or amount of oil used for the intervention?

**Response**: We do not expect any differences in the behavior of the participants with regards to newborn care practices other than skin care – as we took meticulous care in ensuring that intervention workers promoted only the oil and recommended skin care practices and did not touch upon any other care practices. Further, intervention worker visits and evaluation worker visits were scheduled on the same days.

With regard to skin care practices however, the intervention should certainly have led to differences, since there were specific recommendations around the gentleness of massage, quantity of oil to be used, and number of times (ie. three) that massage ought to be performed.

As mentioned in an earlier response, due to expected variance in respondents’ perception of their own massage technique in terms of how gentle it was, we found it best to avoid such a question.

With regards to quantity of oil used per massage, it was difficult to reliably measure and assess whether this differed between intervention and control arms. In the control arm, the usual practice is to store oil in small bowls for use in massage, and reuse leftover oil for subsequent applications. In the intervention arm, we instructed families not to transfer the oil into any bowl, and instead pour the oil straight from the bottle into their palm for application. We also received anecdotal reports that the SSO was much more easily absorbed into the skin and disappeared post application, as compared to mustard oil, that left the skin somewhat sticky post massage.

We did collect information on number of daily applications, however, in the second and third postnatal visits. The difference between mean number of oil applications was small but significant in both the visits, with higher application in the intervention group. Based on the second visit recall, intervention infants were massaged (regardless of oil used) for a mean of 2.68 (95% CI 2.66 – 2.70) times [median and IQR 3±1], whereas control infants were massaged for a mean of 2.38 (95% CI 2.37 – 2.40) times [median and IQR 2±1]. Based on the third visit recall, intervention infants were massaged (regardless of oil used) for a mean of 2.66 (95% CI 2.65 – 2.68) times [median and IQR 3±1], whereas control infants were massaged for a mean of 2.37 (95% CI 2.36 – 2.39) times [median and IQR 2±1].

The following text was inserted into the Methods regarding intervention workers: “Recognizing that there were no corresponding visits comparable to intervention worker visits in the control arm, intervention workers received no training on newborn care and were expressly instructed to refrain from counselling families on other aspects of newborn care besides the intervention itself.”

The following text was inserted into the Methods section under the ‘Evaluation’ head: “We collected data on skin products used and frequency of massage, but did not have reliable means of assessing other practice outcomes such as gentleness of massage technique and amount of oil applied.”

Further, we have created a separate section on massage practices in results, with the following text added: “Frequency of massage: Based on respondent recall from the second evaluation visit, intervention infants were massaged (regardless of oil used) for a mean of 2.68 (95% CI 2.66 – 2.70) times [median and IQR 3±1], whereas control infants were massaged for a mean of 2.38 (95% CI 2.37 – 2.40) times [median and IQR 2±1]. Based on the third visit recall, intervention infants were massaged (regardless of oil used) for a mean of 2.66 (95% CI 2.65 – 2.68) times [median and IQR 3±1], whereas control infants were massaged for a mean of 2.37 (95% CI 2.36 – 2.39) times [median and IQR 2±1].”

6. Was gestational age information collected? If so, please report it for the various groups listed in Tables 1 and 2.

**Response**: It was not possible to conduct ultrasound examinations for accurate dating of gestational age. We did collect the date of last menstrual period, however the distribution of gestational age that we got based on the LMP date appeared unreliable (see table below), giving the rate of preterm birth as ~30% as compared to accepted figures of 13% in South Asia – we therefore felt that it would not be appropriate to use this variable as a reliable measure of gestational age. Biophysical measures for gestational age dating by the data collectors were also not feasible to standardize among 160 workers.

| Gestational age category | Frequency | Percent |
| --- | --- | --- |
| <28 weeks (extremely preterm) | 0 | 0.0% |
| 28-31 weeks (very preterm) | 1,345 | 4.9% |
| 32-36 weeks (moderately preterm) | 7,031 | 25.5% |
| 37-41 weeks (term) | 16,087 | 58.3% |
| >=42 weeks (post-term) | 3,107 | 11.3% |

Thus, we relied on measures of weight, which was well-standardized among the data collectors. This has been further clarified in the Methods with the addition of the text shown here in italics: “Sub-group analyses for NMR were pre-specified based on prematurity, low birthweight (LBW), birthplace, wealth and emollient use. Methods for intention-to-treat analyses were replicated in sub-groups of infants based on 1) prematurity: first visit weight ≤ 1500 g (i.e., very low birth weight, VLBW) [14] as a proxy for very preterm in the absence of ultrasound reports and reliable data from recall of last menstrual period*, i.e., in the absence of reliable measures of gestational age*,…”

7. Please clarify the process that was used to document the use of the oil type in both arms. How was it determined that SSO was used exclusively by 30.4% of subjects and mustard oil by 36%? What is the expected reliability of this method?

**Response**: At the three data collection visits (as soon as possible after birth, around day 7, around day 28), respondents were asked to identify the various products that they applied on the infant’s skin on a typical day from the last visit up to the current visit. Thus, the recall period was about 2-3 days for the first visit (birth to day 2), 5-6 days for the second visit (about day 2 to day 7), and 3 weeks for the third visit. Exclusive SSO/ mustard oil use was subsequently generated as a composite variable that combined these responses across time till the last visit or case closure due to death. The text in italics has been added to the Methods: “*Newborn infants were enrolled and delivery details, including date and time of delivery*, were recorded during postnatal visitation scheduled on day one. Data on newborn survival status, date and time of death if applicable, and oil application *focusing on the time between visits*, were collected during postnatal visitations on days one (*recall period from birth to the first postnatal visit*), seven (*recall period from the first to the second visit*) and 29 after delivery (*recall period from the second to the third visit*).” Data collectors were carefully trained and supervised, and spot checks (5%) and back-checks (5%) were conducted as described in the Methods. However, we would like to clarify that these variables were based on respondent recall and not direct observation. Therefore, the reliability of this method of determining exclusive SSO/ mustard oil use is limited by the recall ability of respondents.

8. The measurement of weight approximated birthweight, as indicated. Were there records for those who delivered with a professional that would have birthweight? Discuss how the weight at enrollment impacted the design or analysis, i.e., how would weight loss or gain between birth and start of study procedures (for intervention) impact outcome and be addressed?

**Response**: Although nearly 85% of deliveries were conducted within health facilities, babies were typically not being weighed at birth and hospital records when examined, were found to be highly inaccurate with unacceptable heaping and error rates. Our data collectors were well trained, standardized and supervised in measurement of birth weight, using calibrated scales. The procedure was comparable for all infants, regardless of group assignment. There was on average about a 2-day period from birth to weighing, and thus the weights that we report may be slightly lower than actual birth weight. However, this would not affect our impact outcome results, except for the subgroup analyses (≤ 2500 g, ≤ 1500 g) which may include a small percentage of infants whose birthweight was above those cutoffs. The following text has been added to the Discussion limitations: “*It was not feasible to reliably capture infant weight at the time of birth and thus we used weight at the first postnatal visit as a proxy for birthweight. There was on average about a 2-day period from birth to weighing, and the weights that we recorded may be slightly lower than actual birth weight (Knowles V et al.* [*http://dx.doi.org/10.1136/archdischild-2020-319158*](http://dx.doi.org/10.1136/archdischild-2020-319158)*). Thus, subgroup analyses (≤ 2500 g, ≤ 1500 g) may include small percentages of infants whose birthweights were above those cutoffs at the time of birth, although this would be expected to have minimal to no impact on the overall findings*.”

Results

1. Please provide demographic information for the groups in table 2, including the GA, weights, gender distribution, day of life at enrollment and statistical comparison. Table 2 currently lists the primary outcome only.

**Response**: Table 1 provides this information on population characteristics by intervention and comparison group for both the intention-to-treat and the per protocol analyses. Table 2 provides the numbers of subjects used in each sub-group analysis. All of the analyses, including the sub-group analyses, adjusted for these factors, and therefore already accounts for these factors. Given the importance of sex-disaggregated data, we inserted text (show here in italics) in the Results for males and females separately for the overall analysis by intention to treat [“*Results were similar for male (aOR 0.94, 95% CI 0.79-1.13) and female (aOR 1.00, 95% CI 0.80-1.25) infants]*”; and in the per protocol analysis [“*Sex-disaggregated data showed slightly higher reduction in NMR in male (aOR 0.40, 95% CI 0.27-0.58, p<0.01) compared to female (aOR 0.53, 95% CI 0.35-0.79, p<0.01) infants by regression analysis.*”]

2. Is there value in showing the time from birth to neonatal death for the two groups?

**Response**: This is shown in the survival curves (Figure 3). (We may have misunderstood the question – our apologies if this is the case.)

3. There were cases of skin rash, etc., for each group. Please provide more detail on the nature of these conditions. Were they associated with a particular birthweight or GA?

**Response**: Data was collected on the presence of skin rash/skin reactions/skin infections based on identification by the data collectors or on self-report of the parent/guardian that there was some abnormality of the skin of the newborn, for example the presence of erythematous papules or pustules. This assessment was not aimed at identifying differences in skin condition or skin barrier integrity or function as has been done in previous hospital-based studies (e.g., Nopper AJ, et al. J Pediatr 1996;128(5 Pt 1):660-9; Darmstadt GL, et al. Pediatr Infect Dis J. 2004;23(8):719–25; Darmstadt GL, et al. Pediatr Infect Dis J. 2014;33(11):1124 –7; Salam RA, et al. Arch Dis Child Fetal Neonatal Ed 2015;100(3):F210-5), and in a community-based cohort in Nepal by Summers A, et al. J Perinatol 2018 Jan;38(1):64-70; Summers A, et al. BMC Pediatr. 2019;19(1):512.). Rather, these assessments aimed to identify adverse events in relation to emollient therapy, such as the development of pustules potentially suggestive of skin infection. The presence of these episodes was confirmed by a specialized team of data collectors; however, the diagnosis of these skin reactions was not confirmed. These reactions were not associated with treatment arm, and they were not associated with any particular birthweight category of infants (≤1500 g: 4.6%; 1501-2500 g: 3.2%; >2500 g: 4.1%). Additional information on skin care practices and outcomes will be provided in a subsequent manuscript currently under preparation.

4. Given the importance of skin barrier integrity in neonatal infection, provide the rationale for not including skin assessment as a measure of emollient effects on skin. Skin rash has been reported previously among newborns receiving either sunflower seed oil or mustard oil in a rural community setting (Summers A, et al. J Perinatol. 2018 Jan;38(1):64-70. doi: 10.1038/jp.2017.158 and Summers A, et al. BMC Pediatr. 2019 Dec 23;19(1):512. doi: 10.1186/s12887-019-1871-2). Was rash or miliaria observed in subjects of the present study? How did the skin integrity in the present study compare to previous reports?

**Response**: There was an original intent to measure transepidermal water loss and skin condition during the latter half of the study, but this could not be pursued due to its premature termination. Rash is discussed in our response above, but skin integrity was not systematically assessed as part of the present study.

Previous hospital studies have included the highest risk preterm infants, generally those < 33 weeks gestational age. Studies of skin barrier function have shown that below this gestational age, there is a marked increase in immaturity and permeability of the skin barrier, which is why initial proof-of-concept hospital studies focused on this highly vulnerable age group. The current study in India included all newborns at a population level and thus the study cohort would have been much more highly variable in the degree of skin barrier permeability compared to previous studies conducted among very preterm infants in hospital settings. However, our cohort in India would likely be similar to the study cohort of Summers et al (J Perinatol. 2018 Jan;38(1):64-70 and BMC Pediatr. 2019 Dec 23;19(1):512). We are unable to systematically compare results from our study with the Nepal studies due to lack of data on these measures.

5. In Table 2, section C, please specify the symbol in the title (§) as it is not currently defined.

**Response**: This has been corrected.

Discussion

1. On page 20, in the paragraph beginning in line 400, include a discussion of the information in the previous report by Summers, A., et al, that describes the effect of mustard versus sunflower seed oil for routine repetitive neonatal skin massage (Summers A, et al. BMC Pediatr. 2019 Dec 23;19(1):512. doi: 10.1186/s12887-019-1871-2) Summers A, et al. BMC Pediatr. 2019 Dec 23;19(1):512. doi: 10.1186/s12887-019-1871-2.

**Response:** Thank you for pointing these out – this is probably the first evidence related to measures of skin barrier integrity in community conditions, and these results do add to a more nuanced interpretation of the findings. We felt that it would be better to discuss this in a full paragraph within discussion as below (page 28-29 in the revised version):

“However, expected effects of SSO or mustard oil based on observations in controlled laboratory or hospital conditions may not bear out similarly in human infants in uncontrolled community settings. Recent studies from Nepal that for the first time assessed skin barrier integrity in human newborns in community settings did not find major differences between SSO and mustard oil groups [42]. Rate of reduction in skin pH during the first week of life was higher in infants massaged with SSO as compared to mustard oil, suggesting a faster acid mantle development and improved barrier normalization, but the improvements were modest [42]. No difference was observed in transepidermal water loss or skin scores and infants massaged with mustard oil fared slightly better in terms of a proxy measure for stratum corneum cohesion [42]. However, unlike previous hospital studies that involved gentle applications of SSO, infants in these studies from both the oil groups were massaged vigorously several times a day, and it is possible that massage technique may have influenced study outcomes [41, 42]. The study was limited by its inability to resolve the independent effects of the oils, massage practices, and environmental conditions [41, 42].”

2. Given that mustard oil was used exclusively 36% of the time in the control group, what was the usage of mustard oil exclusively in the intervention group and how would this impact the conclusions?

**Response**: Among 13,478 infants in the intervention group, a small proportion of 592 infants (4.4%) exclusively used mustard oil, of which 208 died (29.0% of the total number of deaths in the intervention group). In contrast, 4,096 infants (30.4%) exclusively used SSO, of which 256 died (35.7% of total intervention deaths). It is likely that this highly skewed proportion of deaths in the exclusive mustard sub-group in the intervention arm may have contributed towards skewing the overall results of the study in the direction of the null result. However, there are many potential sources of bias, and therefore we cannot confirm if the skewed deaths in the sub-group are attributable to exclusive use of mustard oil.

3. What were the “other” oils used by subjects in either arm when they did not use mustard oil or sunflower seed oil? Is it possible to comment on the range of oils used, along with their expected compositions? How much variation in fatty acid content of various types is to be expected within this population and/or region in India?

**Response**: At the initial skin cleansing after birth and for the first application to the newborn within the first 6 hours of birth, about half of families applied nothing to the skin. In addition to SSO and mustard oil, small minorities of families (<5%) used flour dough, olive oil, or Johnson’s Baby Oil; use of other oils such as a local product (Dabur Lal Tel), sesame oil and coconut oil was rare. In some cases, however, the family applied something besides SSO or mustard oil but could not identify it. We found that families typically stick to mustard oil (and in case of our intervention, SSO) during the first 2 week, and start introducing other oils beyond this period.

We report on growth and morbidity outcomes – secondary outcomes of the study – in another manuscript that is under preparation. In that paper, we provide more detailed information about adherence to recommended practices, including elimination of the practice of bukwa (a mix of oil and pulverized grains) application.

Olive oil is relatively low in linoleic acid and in studies in mouse models it delayed skin barrier recovery as assessed by transepidermal water loss (Darmstadt GL, et al. Acta Paediatr 2002;91:546-554; Elias PM. The Lanolin Book Hamburg, Germany: Beiersdorf AG. 1999:253-79). Sesame oil is relatively high in linoleic acid and coconut oil has shown beneficial effects on infection prevention and promotion of growth in hospitalized preterm infants in Pakistan (Salam RA, et al. Arch Dis Child Fetal Neonatal Ed. 2015; 100(3):F210-5). Sesame oil tends to be sticky and in prior formative research in Bangladesh (similar cultural setting as in North India) we found that it was not popular among families. Johnson’s Baby Oil is comprised of mineral oil.

4. What is the reason for the increase in exclusive adherence from 15.2% to 38.7% later in the study?

**Response**:

Thank you for bringing attention to this aspect – we had omitted certain text that explained this in detail from our original submission in order to cut down on the number of words. However, we think that it would be important to put these explanations back in for a more comprehensive and deeper understanding of the study, in terms of the challenges of shaping newborn care behaviors – especially as part of research when both interventions are assumed to be in equipoise, and the intervention needs to be ethically promoted in the absence of *a priori* conclusive evidence.

It must be appreciated that the community being studied has a very strong belief in the benefits of mustard oil for infant massage that has been passed down from generations. Mustard oil is seen as synonymous with toughness, and it is commonly believed to strengthen the bones of infants. This formative research finding should be generalisable to the entire tracts of regions with high prevalence of mustard oil massage.

We did not deem it appropriate and ethical to directly attack or even dent this widely held notion for several, mostly ethical, reasons. The first and primary ethical consideration was that there was no conclusive evidence on skin barrier impairment due to mustard oil in human infants and previous hospital-based studies had only evaluated SSO vs. no emollient. Secondly, denting the community’s deeply held trust in mustard oil would have placed on us the ethical responsibility of ensuring its replacement post the newborn period and post the completion of the study at a large scale – this was neither feasible, nor ethical in the absence of evidence. Finally, from an implementation perspective, we felt that pitching SSO directly against mustard oil in any way would adversely affect its adoption.

Our behavior change strategy was therefore very carefully designed and co-developed with community members. Our core strategy involved:

1. Sensitizing families to the fragility of the newborn skin: As part of behavior change interactions, intervention workers discussed the difference between the skin of newborns and older infants by showing families a new leaf (akin to a newborn’s skin) from a plant and comparing it to a mature leaf (akin to the skin of older infants). They demonstrated the new leaf as soft and more easily damaged as compared to the mature leaf, which was stronger and more resilient to damage. The newborn’s skin was positioned as analogous to the new leaf, or a flower petal, that was extremely fragile, needing gentle touch and use of non-abrasive ‘soft’ skin products. Families were therefore recommended that they **defer, rather than replace** practices like vigorous massage with mustard oil and application of a homemade scrub called ‘*bukwa*’ until after the newborn period.
2. Positioning SSO as a gentle high-quality oil: The SSO was branded as “Saksham Sneha Oil” (‘saksham’ refers to empowering and ‘sneha’ refers to love) and packaged into attractive amber bottles with a golden cap and a beautifully designed label containing instructions for use. Thus, a new niche was created for SSO as a newborn focused oil that strengthens the fragile newborn skin.
3. Emphasis on early, only, all: i.e. ensuring early application of SSO beginning with the first application (usually right after birth in the labor room itself), ensuring exclusive application of SSO throughout the newborn period, and ensuring these for all newborns, (basically making sure that we are able to all eligible babies in a timely manner).

This was a large trial, and enrolment of study villages into the trial was phased over a period of 6 months in the initial period. Our ~80 intervention workers were typically fresh recruits from the community who had little or no prior work experience, and were trained to promote SSO use amongst intervention families, conduct community meetings, etc. Multiple reasons could have contributed to the increased adherence with time:

- Improved confidence and competence in intervention workers in negotiating behaviour change with families over a period of time.
- Improved quality of support to intervention workers with time: our ~80 intervention workers were spread over 2000 sq kms., and during the middle of the study, we also began to provide them targeted support through a remote teleguidance center that called each worker at least once in 2 days to follow-up on their visits, understand their challenges, and help resolve field issues.
- It was a common practice that families insisted on the vernix being removed in the labour room prior to handing over the baby to them. There was an implicit understanding between facility staff and families that the families would bring their own emollient which would be used by the facility staff to wipe the vernix off the baby’s skin. We realized that this practice of removing the vernix was too deeply entrenched, and difficult to change, but we should instead focus on SSO being used to clean the vernix for the intervention babies. We therefore worked with ASHAs (community health workers who accompany mothers to health facilities for delivery) to ensure that families carried the oil that was distributed to them, when the mother is taken to the facility for delivery. We realized during the early months that families sometimes forgot to carry the oil bottle with them, so we also began to ensure a basic spare stock of oil with the ASHA workers.
- Acceptance of a new product, especially with newborns, needs trust and a good track record of word-of-mouth experience sharing. It therefore takes time. Towards this end, besides promotion of the oil to families through interpersonal communication, we also oriented community masseuses (called nauns) and conducted monthly community meetings with all village stakeholders.

With regards to changes in the manuscript, we felt it was also important to put the low reported adherence to exclusive use of SSO in the intervention arm (30.4%) into perspective against the overall high acceptance of the oil – 89.3% families in the intervention arm reported applying any SSO during the newborn period. This has been included in several places, including the abstract, summary, results and discussion.

We also introduced additional detail on the formative research and behavior change strategy in the methods section as below:

“There was a very strong intrinsic belief in the goodness of mustard oil and its ability to strengthen the bones of infants.”

“Our behavior change strategy emphasized early and exclusive use of SSO, and non-use of any other products or emollients on the skin for all newborns, regardless of birth weight. We did not want to attack the community’s belief in the goodness of mustard oil, and therefore, our behavior change strategy simply promoted SSO as an oil that was suited to the fragile skin of the newborn, which was like the petal of a flower, and asked them to defer application of mustard oil and homemade scrub ‘bukwa’, until after the newborn period.”

We have added the following text to the discussion:

“The community’s inherent belief in the goodness of mustard oil may have prevented them from giving up entirely on its use during the newborn period, while it may have been easier for them to accept a new oil for newborn massage that also appeared to be good. We did not consider it ethical to attack this pre-existing belief in mustard oil in the absence of conclusive evidence of its inferiority in human newborns and also our inability to provide them with a continuous supply of SSO beyond the newborn period and beyond the duration of the study.” and (additional text italicized) “Adherence to exclusive SSO improved from 15.2% in June 2015 when implementation was rolled out in all clusters to 38.7% in September 2016 when enrolment was suspended, *reflecting the need to allow sufficient time to shift deeply entrenched community practices in studies of this nature*.”

5. On page 21, in the paragraph starting with line 430, the authors discuss that the study population who were subject to mortality due to skin barrier compromise may have been too low. What is the basis for this statement? If there is information about the relationship between birthweight or gestational age, day of life, skin barrier integrity/compromise and mortality due to compromise, please elaborate in this section and include the references. Similarly, provide information about the percent of the population that was considered normal term intra-uterine growth restricted. It would be of interest to know more details about the two populations in terms of growth restriction and percentage of infants at various weights or gestational ages.

**Response**: We state the following: “…level of vulnerability of the study population to mortality due to skin barrier compromise may have been too low overall to measure population-level impact.” Note that due to lack of reliable gestational age measurement, we cannot determine rates of intrauterine growth restriction. However, other studies have shown that in South Asian contexts, sizeable proportions of LBW infants are term-small-for-gestational age. The text that follows has been modified to provide further explanation, with added text in italics: “*As prematurity and low birth weight becomes more extreme, the skin barrier is increasingly immature, easily damaged, and functionally compromised (Hammarlund K, et al.* Acta Paediatr Scand 1979;68: 795–801; Kalia Y, et al. J Invest Dermatol 1998; 111: 320–26; others)*. Further, this is associated with increased risk of mortality, due in part to accelerated losses of water and heat through the skin and compromised skin barrier protection again the entry of pathogens through the skin (Darmstadt GL et al. Pediatr Res. 2007 May;61(5 Pt 1):588-93; Choi Y, et al. Neonatology. 2008;94(2):123-31).* The infants most vulnerable to skin barrier compromise – those who were VLBW and also likely to be very preterm – constituted only 2.2% of the study population and 5.2% of deaths *in our study – too few to drive measurable change in population-level mortality on their own*. *The next most vulnerable category of* LBW infants *weighing 1501-2500 g*, while a sizeable 38.6% (n=10,269) of *our study* population, *is typically comprised of* a significant proportion of term intra-uterine growth restricted infants *in the South Asian* *context* (Lee ACC, et al. Lancet Glob Health 2013 Jul;1(1):e26-36; Kozuki N, et al. J Nutr 2017;147(Suppl):2141S–6S.) whose skin-barrier function is relatively unimpaired [Hammarlund K, et al. Acta Paediatr Scand. 1980 May;69(3):377-83; Hammarlund K, et al. Acta Paediatr Scand. 1983 Sep;72(5):721). *This group had 32.1% of the deaths in the study population, reflecting their relatively low vulnerability compared to the VLBW infants (Table 2).*

1. On page 21, in lines 439-441, there is a statement that mortality was 52% reduced in infants ≤ 1500 g, despite low adherence to SSO. What are the potential explanations for this finding? Does the statement indicate that the mortality was reduced enough in the infants who adhered to the treatment to account for the reduction in the total group? Please clarify.

**Response**:

We believe that the morality reduction in infants ≤ 1500 g could be due to 2 main reasons: (a) greater impact of exclusive application of SSO (i.e. per protocol), and (b) greater impact even of non-exclusive application of SSO (i.e. even if there was a single application) - the premise being that these vulnerable infants preferentially benefitted from any application of SSO. The sample size is small, but in the intervention group, of a total of 311 VLBW infants, 239 infants received any application of SSO, vs. 72 that did not receive any SSO. Neonatal deaths in the former were 34 (14.2%) as compared to 44 (61.1%) in the latter, giving a chi-square statistic of 64.7 with p<0.0001. We have added explanatory text as below: “Sub-group analysis which focused on the most vulnerable VLBW infants ≤ 1500 g found a significant 52% reduction in mortality in the SSO arm. Adherence to recommended treatment of exclusive SSO application in this subgroup was particularly low (23.5% overall), *however 76.8% of VLBW intervention infants received SSO at least once vs. 1.8% of infants in the comparison group. This reflects the possibility of enhanced benefit from SSO therapy, even with partial adherence, in infants with the highest level of skin barrier compromise and vulnerability.*”

2. On page 24, in the paragraph starting with 505, include a discussion of the effects of mustard oil massage in the immediate newborn period in Nepal.

**Response:** Please note our response to your first comment in the Discussion section as below. This has been addressed as an additional paragraph in page 28-29 in the revised document. “However, expected effects of SSO or mustard oil based on observations in controlled laboratory or hospital conditions may not bear out similarly in human infants in uncontrolled community settings. Recent studies from Nepal that for the first time assessed skin barrier integrity in human newborns in community settings did not find major differences between SSO and mustard oil groups [42]. Rate of reduction in skin pH during the first week of life was higher in infants massaged with SSO as compared to mustard oil, suggesting a faster acid mantle development and improved barrier normalization, but the improvements were modest [42]. No difference was observed in transepidermal water loss or skin scores and infants massaged with mustard oil fared slightly better in terms of a proxy measure for stratum corneum cohesion [42]. However, unlike previous hospital studies that involved gentle applications of SSO, infants in these studies from both the oil groups were massaged vigorously several times a day, and it is possible that massage technique may have influenced study outcomes [41, 42]. The study was limited by its inability to resolve the independent effects of the oils, massage practices, and environmental conditions [41, 42].”

**Reviewer #2**: Your article is very well written. It is a valuable work and it will definitely be very useful for your community.

It has no methodological, scientific or executive problems. I have no comments in these areas.

There is only one problem : this intervention is known to some extent and all researchers recognize its effectiveness. What kind of innovation or difference is in your activity and research that distinguishes it from others? Please include the importance of study and its necessity  in the introduction section.

It may still be a questionable problem or have a particular importance to your community.

Best regards,

**Response**: The following text was added to the Introduction: “*Unlike previous hospital-based studies in preterm infants, this trial evaluated emollient therapy application on all newborns by families (rather than health workers) in a community setting across the facility-community continuum, and with usual skin care practices (rather than no emollient application) as the comparison* and with a primary outcome of mortality*.*”

**Reviewer #3**: This is a well-conducted Cluster RCT on the impact of emollient therapy with sunflower seed oil on survival of newborn infants in Uttar Pradesh, India. The study design, sample size, statistical methods and analyses, and presentation (tables and figures) and interpretation of the results are mostly adequate and of a good standard. However, there are still a few issues needing attention.

1) Need to make it absolutely clear the study uses co-primary outcomes including both NMR and post-24-hour NMR.

**Response**: The NMR and post-24-hour NMR outcomes were not considered as co-primary, as we did not hypothesize that both of them should be significantly reduced in order to conclude if the intervention had any impact. We had expected both these outcomes to have the same direction. Further, we added the post-24-hour NMR outcome only to potentially improve the power of the study, assuming (perhaps incorrectly), that first/ early application would not impact deaths within the first 24 hours. We have added the word “independent” to clarify this point: “NMR and post-24-hour NMR were *independent* primary outcomes of the trial. Post-24-hour NMR was included as a primary outcome as we assumed that the intervention was unlikely to have an effect on deaths within the first 24 hours and including this outcome could potentially improve the power of the study.”

2) Sample size section on page 6. Need a bit more details and clarification how the sample size was calculated as need to satisfy both co-primary outcomes so may need to calculate separately and then combine and choose one sample size for both. Need justification/reference for all the parameters used for the sample size calculation. Why ICC is 0.1? Why assumed a mean NMR of 45 per 1000? and etc.

**Response**: The details regarding the various assumptions in the sample size calculation may be found in the Trial protocol document. We did calculate the sample size of both the outcomes separately, and the NMR outcome required the larger sample size, which was therefore used for the trial.

The NMR of 45 per 1000 was assumed based on preliminary results from a recent study (‘AMANHI’) conducted in the same area on estimating the burden of maternal and neonatal mortality and stillbirths, with a slightly different surveillance methodology. (See <https://www.thelancet.com/journals/langlo/article/PIIS2214-109X(18)30385-1/fulltext> )

The between-cluster variation ‘k’ of 0.1 was based on an expected NMR range of ±15% in the control clusters. This is somewhat lower than ‘k’ values of 0.12-0.16 for NMR observed in some other studies from South Asia. (<https://trialsjournal.biomedcentral.com/articles/10.1186/1745-6215-12-151> )

The number of births in each cluster (149) was calculated using mean cluster population of 3000, crude birth rate of 27 per 1000 population per year, 2 years of enrollment per cluster, and 8% loss to follow-up.

We have accordingly amended the sample size section (edits italicised), referencing the trial protocol document and the AMANHI study as follows:

“The sample size was estimated to enable detection in intention-to-treat analysis of at least 15% reduction in NMR and 20% reduction in post-24-hour NMR at 5% level of significance with 90% power [17]. *A mean NMR of 45 per 1000 was assumed [18], with a range of ±15% across control clusters, yielding a* between-cluster coefficient of variation of 0.10 *[17].* Calculations assumed 162 births per cluster (*mean* population ~3000 *and crude birth rate of 27 per 1000 over an enrolment period of 24 months),* expected loss to follow-up of 8%, yielding a sample size of 138 clusters per study arm (or 20,536 newborn infants in each arm) *for NMR as the primary outcome. The sample size calculated for post-24-hour NMR was lower at 132 clusters [16], and therefore n=276 clusters was taken as the final sample size.*”

3) Primary analysis on page 10-11. It's a bit confusing as both logistical regression and survival analysis (proportional hazard model) were used. There can only be one primary analysis, and other one (survival) need to be toned down as sensitivity check. The results are both negative, otherwise (one negative, one positive) will be confusing and inconsistent.

**Response**: Thank you for seeking this clarification. Our primary analysis was based on intention-to-treat regression analysis, and this has been clearly labelled in the methods section. Within the section on primary analysis, we have also now clearly stated that the survival analysis was done as a sensitivity analysis: “We also estimated the treatment effect on survival time, *as a sensitivity analysis*, using Kaplan-Meier curves (taking day 0 as the day of birth and day 27 as the 28th completed day of the neonatal period) and performed proportional hazards regression adjusted for clustering and the covariates listed above.”. We have also reiterated this in the results section as below: “*Sensitivity analysis based on survival time* also showed no difference in survival of newborn infants in the intervention vs the comparison group…”

4) Table 1. Some of the continuous variables are not normally distribution (skewed) such as cluster size and age at measurement of first visit weight, therefore need to present as median and IQR rather than mean and SD.

**Response**: The cluster size and age at measurement of first visit weight for both control and Intervention are non-normally distributed, hence median and IQR has been calculated, and have been updated in Table 1.

Cluster size: Control 80±53; Intervention 82.5±60

Age at measurement of first visit weight: Control: 2±2; Intervention 2±2
