# Supplementary material for: Effect of sunflower seed oil emollient therapy on newborn infant survival in Uttar Pradesh, India: A community-based, cluster randomized, open-label controlled trial
Source: PLoS Med. 2021 Sep 28;18(9):e1003680. doi: 10.1371/journal.pmed.1003680 (PMC8478176; doi:10.1371/journal.pmed.1003680)
Supplement: Attachment — Submitted filename: Manuscript PMEDICINE-D-20-04871R2.docx [file pmed.1003680.s005.docx]

Manuscript PMEDICINE-D-20-04871R2: Impact of emollient therapy with sunflower seed oil on survival of newborn infants in Uttar Pradesh, India: a community-based, cluster randomized, open-label controlled trial.

Reviewer Comments

Overall

In general, the authors have addressed the comments satisfactorily both in their response to them and in revisions to the manuscript.

Specific Comments

1. The trial was designed to reflect effects of emollient therapy in rural settings. Table 1 indicates that about 15% of babies were delivered at home. Of interest are the following: Is the distribution of home versus facilities as the birth place typical for the region? Were the facilities mainly hospitals with comparable levels of service? What is the typical time spent in the facility by mother and baby? Were there any babies, e.g., very premature, who stayed in the facilities for some form of specialized care? For babies born in facilities, who conducted the massage and emollient procedures right after birth and over the first day?
2. In the methods, study design and sample, there is a description of the use of post-24-hour NMR as an outcome. The statement is made that “assuming that the intervention was unlikely to impact mortality within the first 24 hours”. What is the basis of this statement? As stated elsewhere in the paper, the population ≤ 1500 g is expected to have a skin barrier that is easily damaged? Would it not be possible that application of emollient and/or massage would have effects early on? There are reports of rapid barrier development, particularly upon exposure to dry (drier than in utero) conditions.
3. A key finding was that the mortality was significantly lower for the intervention versus the control in infants ≤ 1500 g. Yet, this group was only 2.2% of the total population. There were other confounders, such as the number of infants who used only SSO. The sample size is too small to remove the confounder of the use of multiple products. How would you address the possibility that the presumably “more vigorous” massage in the control group would not impact the mortality rate? Consider modifying the discussion to recognize that the oil type and massage type cannot be separated.
4. Please comment on whether there were differences for control and intervention for the time from birth until death. How did the NMR in this study compare with overall reports for the region during the study period?
5. Was any information collected regarding the cause of death for control and intervention? The basis is that mortality may occur because of an immature barrier and subsequent infection. Were there any measures of the infection rates for the two populations?
