# Supplementary material for: Effect of sunflower seed oil emollient therapy on newborn infant survival in Uttar Pradesh, India: A community-based, cluster randomized, open-label controlled trial
Source: PLoS Med. 2021 Sep 28;18(9):e1003680. doi: 10.1371/journal.pmed.1003680 (PMC8478176; doi:10.1371/journal.pmed.1003680)
Supplement: Attachment — Submitted filename: PMEDICINE-D-20-04871R2_Response_21March.docx [file pmed.1003680.s006.docx]

**PMEDICINE-D-20-04871R2 Response to editors’ & reviewers’ comments**

Dear editors,

Thank you very much for the helpful editorial and reviewer comments. We have provided our responses below and look forward to your review.

Sincere regards,

Dr. Vishwajeet Kumar

Figures: In addition, we request that you upload any figures associated with your paper as individual TIF or EPS files with 300dpi resolution at resubmission; please read our figure guidelines for more information on our requirements: http://journals.plos.org/plosmedicine/s/figures. While revising your submission, please upload your figure files to the PACE digital diagnostic tool, https://pacev2.apexcovantage.com/. PACE helps ensure that figures meet PLOS requirements. To use PACE, you must first register as a user. Then, login and navigate to the UPLOAD tab, where you will find detailed instructions on how to use the tool.

Response: We have followed this procedure to resubmit the final figures.

We ask every co-author listed on the manuscript to fill in a contributing author statement, making sure to declare all competing interests.

Response: This is done.

To enhance the reproducibility of your results, we recommend that you deposit your laboratory protocols in protocols.io, where a protocol can be assigned its own identifier (DOI) such that it can be cited independently in the future.

Response: We tried using protocols.io, but it appears to be designed more for laboratory protocols. Our protocol is currently available in the Harvard Dataverse, at <https://doi.org/10.7910/DVN/TGNC9H>. Please let us know if you would like us to still try to upload the study protocol on protocols.io

Please ensure that the paper adheres to the PLOS Data Availability Policy (see http://journals.plos.org/plosmedicine/s/data-availability), which requires that all data underlying the study's findings be provided in a repository or as Supporting Information.

Response: Our manuscript adheres to the PLOS Data Availability Policy. Additionally, we have reworded the ‘Data Availability’ section in the manuscript as follows: “The data that supports the findings of this study and data dictionary are openly available in Harvard Dataverse, V1 at <https://doi.org/10.7910/DVN/TGNC9H>.”

Requests from the editors:

1. Abstract: Methods and findings: Please note which variables were adjusted for in reporting the adjusted odds ratios for the primary outcome.

Response: This has been done. We have inserted the following line in the Methods and findings section of the abstract: “Regression analysis was adjusted for caste, first-visit weight, delivery attendant, gravidity, maternal age, maternal education, sex of the infant, and multiple births.”

2. Throughout: Please use consistent terms for children (“newborn infant” vs. “babies” etc.)

Response: This is done – we have consistently used the term newborn infant.

3. Author summary: Why was this study done?: We suggest combining/shortening some of the bullet points in this section- for example, points 2 and 3 could be combined into a single point briefly describing the background/rationale for the study, and the final point could more succinctly describe the study objective/hypothesis.

Response: We have attempted to considerably simplify the entire author summary. We simplified this section into 3 bullet points as follows -
**Why Was This Study Done?**

- Preterm infants have an immature skin barrier, making them susceptible to systemic infections. In animal models, some emollients such as sunflower seed oil (SSO) were found to enhance and mustard oil was found to impair skin barrier integrity.
- Hospital-based studies with emollient therapy in preterm infants demonstrated 50% reduction in bloodstream-proven infections and 27% reduction in neonatal mortality rate (NMR).
- We hypothesized that in community settings in South Asia with near-universal prevalence of vigorous massage with mustard oil, all newborn infants, regardless of maturity, may be susceptible to impaired skin barrier integrity, and therefore may benefit from emollient therapy with SSO, leading to reduction in NMR.

4. Author summary: What did the researchers do and find? Again, for this section, we suggest fewer- potentially three bullet points: 1) What was done; 2) primary findings; 3) secondary findings.

Response: We have attempted to simplify this section into 3 bullet points as follows –

**What Did the Researchers Do and Find?**

- We conducted a community-based cluster randomized controlled trial to evaluate the impact of SSO therapy as compared to usual massage practices in newborn infants.
- Primary intention-to-treat analysis found no difference in NMR between the intervention and control arms.
- Secondary analysis found 52% lower NMR in the sub-group of very low birth weight infants (≤ 1500 g), and 58% lower NMR in all newborn infants treated exclusively with SSO in intervention clusters compared to those who exclusively received mustard oil massage in the comparison clusters.

5. Author summary: What do these findings mean? We suggest combining the first three points into 1-2 points as follows:

-Our findings do not support recommendations for universal promotion of SSO therapy under typical implementation conditions for mortality reduction. However, the low levels of adherence to recommended treatment and a significant mortality reduction by per protocol analysis that we observed suggests that a potential benefit of SSO over mustard oil cannot be ruled out and needs systematic investigation.

-Results from our sub-group analysis on the highest-risk category of very low birth weight infants are consistent with previous hospital-based studies and potentially extend the applicability of emollient therapy in this sub-group to the facility community Continuum.

Response: Thank you. We have attempted to simplify this as follows:

**What Do These Findings Mean?**

- Our primary findings do not support recommendations for universal promotion of SSO therapy for NMR reduction in community settings.
- Secondary findings in very low birth weight infants appear consistent with previous hospital-based studies and suggest possible benefit of extending emollient therapy in this sub-group along the facility-community continuum, which needs to be further evaluated through an appropriately designed study.

6. Methods: Please add the following statement, or similar, to the Methods: "This study is reported as per the Consolidated Standards of Reporting Trials (CONSORT) guideline (S1 Checklist)."

Response: This is done.

7. Methods: On page 15 of the Methods, a predetermined analysis plan is mentioned. Please include your analysis plan as a supporting information file, noting any instances where the analyses conducted were different from those outlined.

Response: We have included a supporting file called “Prespecified analysis plan and modifications”.

8. Results: Page 19: Frequency of massage: Please indicate if the times reported are in times/day or other unit (“...intervention infants were massaged (regardless of oil used) for a mean of 2.68 (95% CI 2.66– 2.70) times [median and IQR 3±1]...”)

Response: This is updated as follows:

*Frequency of massage:* Based on respondent recall from the second evaluation visit, intervention infants were massaged (regardless of oil used) for a mean of 2.68 (95% CI 2.66 – 2.70) times/day [median and IQR 3 (2-3)], whereas control infants were massaged for a mean of 2.38 (95% CI 2.37 – 2.40) times/day [median and IQR 2 (2-3)]. Based on the third visit recall, intervention infants were massaged (regardless of oil used) for a mean of 2.66 (95% CI 2.65 – 2.68) times/day [median and IQR 3 (2-3)], whereas control infants were massaged for a mean of 2.37 (95% CI 2.36 – 2.39) times/day [median and IQR 2 (2-3)].

9. Results: Page 20: Intention to treat analysis of NMR: Please provide units for the NMR values reported here (“...did not find a significant difference in mortality among newborn infants in the intervention (NMR 53.7 ± 2.3)...”

Response: This is updated as follows:

Primary intention-to-treat regression analysis did not find a significant difference in mortality among newborn infants in the intervention (NMR 53.7 ± 2.3 per 1000 livebirths) vs. the comparison group (NMR 56.2 ± 2.4 per 1000 livebirths)

And

Sub-group analyses showed a significant 52% (95% CI 12-74%, p=0.02) reduction in mortality among the subgroup of VLBW infants (2.2% of the study population) in the intervention (NMR 299.3 ± 37.8 per 1000 livebirths) vs. the comparison (NMR 402.4 ± 39.6 per 1000 livebirths) group (Table 2B).

10. Discussion: Page 25: Please revise and clarify this sentence if possible, as the meaning is slightly unclear: “In retrospect, more appropriate study designs than that used here may have been an efficacy trial in all newborn infants which ensured that all infants received the therapy as intended and examined the population-level mortality effect, or a population-based effectiveness trial targeted to highly vulnerable VLBW infants – a group in whom the treatment has shown efficacy under hospital conditions – but under conditions of public health delivery with an eye toward scalability by the health system or potential through markets if the intervention was found promising.”

Response: Thank you for pointing this out. It is indeed a complex sentence. We have removed it as we felt that it was causing confusion without adding commensurate value to the discussion.

11. Table 2: Please also provide the unadjusted results (this may be in a separate/supporting information table if preferred).

Response: We have provided the unadjusted results in an additional column in Table 2.

12. Figure 3: Please note in the legend that the graph in panel B has a different y axis scale than the other two.

Response: We have aligned all axes to a standard 0-1 scale.

13. CONSORT Checklist: Thank you for including the CONSORT checklist, please revise the checklist using section/paragraph numbers to refer to locations in the text, rather than page numbers.

Response: We have revised the CONSORT checklist as advised.

Comments from the reviewers:

Reviewer #1:

Overall

In general, the authors have addressed the comments satisfactorily both in their response to them and in revisions to the manuscript.

Specific Comments

1.	The trial was designed to reflect effects of emollient therapy in rural settings. Table 1 indicates that about 15% of babies were delivered at home. Of interest are the following: Is the distribution of home versus facilities as the birth place typical for the region? Were the facilities mainly hospitals with comparable levels of service? What is the typical time spent in the facility by mother and baby? Were there any babies, e.g., very premature, who stayed in the facilities for some form of specialized care? For babies born in facilities, who conducted the massage and emollient procedures right after birth and over the first day?

Response: Distribution of place of birth: Yes, the distribution of home vs. facility births is typical of the region, and part of an increasing trend towards facility births that has been observed over the last 14 years as a result of an incentivization scheme to promote institutional deliveries. Pregnant women and newborns were recruited into the study through 2-monthly cycles of door-to-door surveillance that aimed to capture all pregnancies and births. The study area covers a district hospital, 10 community health centers, lower level government delivery facilities and other private facilities. Of these, the levels of service among the various community health centers (55.0% of all births) are similar across. No difference was observed in the distribution of place of birth across study arms.

The typical time spent in facilities post delivery ranges from 2-6 hours in most facilities, to 48 hours in the district hospital. We did not collect this information in the present study, but this is based on our work with the health system in the region. For infants born in facilities, it is a common practice to rub the infant with oil brought by the family to remove the vernix. This is typically done by the “dai”, a contractual worker to assists in delivery and immediate newborn care. This is taken to be the “cleaning” or first application. Most families come back within a few hours of delivery, after which the typical home rituals take over. If mothers do stay on for longer in the postnatal care ward, then the oil is typically applied by a family member, though a full massage is usually not provided within the facility.

There definitely would be some babies who would have been referred to the district hospital for care in the sick newborn care unit. However, we did not specifically capture this information, besides the question on hospitalization. Comparison of hospitalization rates does not differ in the two arms, as already mentioned in the paper (Section on Results – Safety, page 21). We would like to also mention that in general, the service quality of newborn care units – both public and private was quite poor in this region during this time period, though quality improvements efforts have been ongoing.

2.	In the methods, study design and sample, there is a description of the use of post-24-hour NMR as an outcome. The statement is made that “assuming that the intervention was unlikely to impact mortality within the first 24 hours”. What is the basis of this statement? As stated elsewhere in the paper, the population ≤ 1500 g is expected to have a skin barrier that is easily damaged? Would it not be possible that application of emollient and/or massage would have effects early on? There are reports of rapid barrier development, particularly upon exposure to dry (drier than in utero) conditions.

Response: We agree that the assumption was erroneous and have mentioned this in the discussion on page 25: “In hindsight, the choice of post-24-hour NMR as one of the primary outcomes of the study was a poor one. Although introduced with the intent to improve the power of the study, the assumption of delay in accrual of benefit was not observed (e.g., Figure 2B). The decision to choose 24 hours as the cut-off point was convenient but arbitrary and not recommended for future studies.”

The reason for including post-24-hour NMR in the first place was the possibility of an increase in power if we were to focus the analysis where most of the mortality impact was likely to be observed. While we understood that skin barrier disruption may occur early, we still did not believe that it could lead to death within the first 24 hours. However, this assumption came into question in the secondary sub-group and per-protocol analysis, as shared above in the discussion section.

3.	A key finding was that the mortality was significantly lower for the intervention versus the control in infants ≤ 1500 g. Yet, this group was only 2.2% of the total population. There were other confounders, such as the number of infants who used only SSO. The sample size is too small to remove the confounder of the use of multiple products. How would you address the possibility that the presumably “more vigorous” massage in the control group would not impact the mortality rate? Consider modifying the discussion to recognize that the oil type and massage type cannot be separated.

Response: We agree that the vigorous massage technique could definitely impact mortality, although we have observed that very small babies are typically just applied oil without vigorous massage. We have already mentioned the following in the discussion on page 26: “The mechanism of impact of SSO therapy on newborn survival is biologically plausible and may be multi-factorial, but requires further research [12,29,30]. The intervention was promoted as a package of SSO plus gentle massage, and thus, we cannot disentangle the separate impacts of the oil from the way in which it was applied. However, it is likely that they interact and that both are important in intervention promotion [29]. Evidence furthermore suggests that emollient therapy has benefits for very preterm infants beyond that provided by massage alone [31-33].”

4.	Please comment on whether there were differences for control and intervention for the time from birth until death. How did the NMR in this study compare with overall reports for the region during the study period?

Response: We had attached the Kaplan-Meier survival curves in figures 3 (a-c) and figure 4, which we describe below. Figure 3a shows the survival curve for all newborns, which appears to be the same in both intervention and control arms. Figure 3b shows the survival curve for the subgroup of <=1,500g infants, which was found to be significantly different [HR 0.65 (0.43-0.97)] between intervention and control, with the effect beginning from the first day itself and predominantly occurring during the first week, with the curves continuing to diverge gradually beyond the first week. Figure 3c shows the survival curve for the subgroup of low birth weight infants, which is not significantly different between intervention and control. Figure 4 shows the survival curve for the per protocol analysis, where we see a significant difference [HR 0.47 (0.35-0.62)] between infants in intervention clusters exclusively applied SSO as recommended vs. those in control clusters applied mustard oil exclusively.

The district fact sheet of Raebareli district derived from sample registration survey data in 2014 mentions the NMR as 33 per 1000 livebirths (http://upnrhm.gov.in/assets/site-files/dhap/districts/Reibareilly/Raibareily.pdf). Over the last decade, there has been an increasing appreciation globally of how different methods lead to different counts of mortality, with retrospective sample-based surveys resulting in a systematic undercount of deaths. Our study followed the most rigorous process of 2-monthly door-to-door surveillance and prospective follow up of pregnant mothers, with key informant-based delivery notification to identify all pregnancies and births in the study clusters. We had conducted the AMANHI study (https://www.thelancet.com/journals/langlo/article/PIIS2214-109X(18)30385-1/fulltext), which was also based on a prospective follow-up just prior to this study, with some geographical overlap with the current study, that yielded an NMR of 41.7 (39.6–43.7). However, in the AMANHI study, we had one surveillance worker covering approx. 15,000 households every 2 months as compared to the present study, where each surveillance worker covered 6-7,000 households every 2 months. This may be the reason for an undercount of births and deaths in the AMANHI study, and the present study really offers the most rigorous NMR count in Uttar Pradesh during the study period.

5.	Was any information collected regarding the cause of death for control and intervention? The basis is that mortality may occur because of an immature barrier and subsequent infection. Were there any measures of the infection rates for the two populations?

Response: Yes, we indeed conduced verbal autopsies based on the WHO instrument which were coded based on a standardized process. Please find the causes of death by study arm for all newborns as well as for <= 1,500g infants below. We conducted a chi-square test to test for differences between the two arms, and did not find a significant difference. We did not collect any other measures of infection rates besides skin rashes and infections, which did not statistically differ in the two arms.

| **Cause of death** | **All newborn infants** | | **<=1500g infants** | |
| --- | --- | --- | --- | --- |
|  | **Control (%)** | **Intervention (%)** | **Control (%)** | **Intervention (%)** |
| Unclassified | 5.37 | 6.56 | 1.05 | 3.85 |
| Congenital malformations | 3.25 | 3.21 | 3.16 | 0 |
| Neonatal diarrhoea | 0.42 | 0.7 | 1.05 | 2.56 |
| Neonatal pneumonia | 3.53 | 3.77 | 1.05 | 1.28 |
| Neonatal Sepsis/ Meningitis | 24.15 | 26.64 | 16.84 | 20.51 |
| Neonatal Tetanus | 0.56 | 0.84 | 1.05 | 0 |
| Perinatal asphyxia | 39.41 | 36.54 | 28.42 | 16.67 |
| Preterm birth complications | 0.14 | 0 | 43.16 | 50 |
| Other specific perinatal causes | 4.94 | 4.18 | 4.21 | 5.13 |
| p-value of chi-square test | 0.86 | | 0.37 | |

Reviewer #3: Many thanks authors for their great effort to improve the manuscript. The authors have addressed my comments very well. I am mostly statisfied with the response and revision. Only one very minor point remaining. On point 4 using median and IQR for non-normal variables (Table 1), it should be presended, for example, 80 (60-113) rather than 80±53 (i don't know exactly the IQR so just give an example). IQR is a range so need to present as medain (xx-yy). Need to correct for both cluster size and age. It's a very minor point. Depending on the editor, as long as it's corrected may not need to come back to me again.

Response: Thanks very much. We have made these changes in the text as well as in Table 1.
