# Supplementary material for: Effect of sunflower seed oil emollient therapy on newborn infant survival in Uttar Pradesh, India: A community-based, cluster randomized, open-label controlled trial
Source: PLoS Med. 2021 Sep 28;18(9):e1003680. doi: 10.1371/journal.pmed.1003680 (PMC8478176; doi:10.1371/journal.pmed.1003680)
Supplement: Attachment — Submitted filename: Comments_Emollient mortality trial_4_May_2021.docx [file pmed.1003680.s007.docx]

| **S.No.** | **Comments** | **Status** |
| --- | --- | --- |
| **Request from editors** | | |
| 1. | Title: Please revise the title slightly, we suggest: “Effect of sunflower seed oil emollient therapy on newborn infant survival in Uttar Pradesh, India: A community-based, cluster randomized, open-label controlled trial” or similar | Done as suggested. |
| 2. | Data availability statement: Thank you for providing a link to access the dataset.  It seems as if download of the data file is restricted.  Please indicate whether this restriction will be removed.  If data are freely available upon request, please note this and state the owner of the data set and contact information for data requests (web or email address). Note that a study author cannot be the contact person for the data | We had planned to remove the restriction on the dataset once the paper is accepted for publication. This is now done. |
| 3. | Abstract: Methods and Findings: Please change to make it completely clear that the numbers here refer to clusters and not infants: “...recommended in intervention clusters (n=138 clusters); infants in comparison clusters (n=138 clusters)...” | Done as recommended |
| 4. | Abstract: Methods and Findings: Please indicate whether you are reporting mean +/- standard deviation: (52.2% male, mean weight 2575.0 grams ± 521.0). | Done |
| 5. | Abstract: Methods and Findings: We suggest revising this sentence to: “...differences between the intervention and comparison groups in other pre-specified subgroup comparisons by low birth weight, birthplace, wealth and emollient use did not reach statistical significance.” or similar to clarify | Done as recommended. |
| 6. | Method: Page 14: Data Management section: Please clarify what is meant by “observed heaping” unless this is a common term | Substituted “distributions of observed values” for “observed heaping” |
| 7. | Methods: Page 14: Statistical analysis section: Thank you for including the supporting information file titled: “Prespecified analysis plan and modifications”.  Please refer to this document in the text of the Methods here, for example as S1_Analysis Plan | Done as recommended |
| 8. | Results: Page 18: Please indicate the numbers in parentheses here are the range of days for the first visit: “At the first postnatal (enrolment) visit that occurred on day 2 (1-3) for…” | Inserted “interquartile range: days 1 - 3” |
| 9. | Discussion: Please ensure the Discussion is organized as follows: a short, clear summary of the article's findings; what the study adds to existing research and where and why the results may differ from previous research; strengths and limitations of the study; implications and next steps for research, clinical practice, and/or public policy; one-paragraph conclusion.  Specifically, on page 26-27, please consider whether the discussion of SSO therapy mechanisms could be discussed earlier in the section. | The Discussion has been modified as suggestion. Additional key results are mentioned at the beginning of the Discussion. Some of the material on pages 26-7 has been moved up to the portion of the Discussion that puts our results into the context of other studies. Some of the material on pages 26-7 has been retained there as it relates primarily to next steps for research. |
| 10. | References: Please check formatting of references 8, 9, 10 (punctuation after journal name).  Please provide an update on reference 31- listed as “submitted”- please note that papers cannot be listed in the reference list until they have been accepted for publication or are publicly available on a preprint archive. Alternatively please provide a different appropriate reference. | Done for references 8,9,10,31. |
| 11. | Table 1: Please define abbreviations “IQR” and “SD” in the legend. | Done as recommended. |
| 12. | Table 2: The footnote symbol for part C (exclusive mustard oil vs exclusive SSO) does not appear to be defined in the legend.  Please check that the notes included in the legend match those shown in the table | The footnote symbol for part C has been removed, as it was an error |
| 13. | Figure 3 and 4: Please note the HR are presented with 95% CIs | This has been clarified by noting that the numbers in the parentheses are 95% CIs. |
| 14. | Checklist/Supporting information: The CONSORT checklist is not included the set of files with the manuscript. Please include the revised version of the checklist (with section/paragraphs rather than page numbers to identify locations within the text) | The section/paragraph locations have been updated and added to the CONSORT checklist |
